# Investigation of the soybean infiltration process utilizing low-field nuclear magnetic resonance technology

**DOI:** 10.1371/journal.pone.0297756

**Published:** 2024-02-16

**Authors:** Lisha Guo, Han Wang, Chenru Hao, Ziqiang Chi, Li Cheng, Haibo Yang, Jing Zhang, Ruibin Zhao, Yanru Wu

**Affiliations:** 1 Department of Medical Physics, School of Medical Imaging, Hebei Medical University, Shijiazhuang, China; 2 Department of Medical Imaging, Hebei General Hospital, Shijiazhuang, China; Tamil Nadu Dr J Jayalalithaa Fisheries University, INDIA

## Abstract

This paper employs low-field nuclear magnetic resonance (LF-NMR) technology to meticulously analyze and explore the intricate soybean infiltration process. The methodology involves immersing soybeans in distilled water, with periodic implementation of Carr-Purcell-Meiboom-Gill (CPMG) pulse sequence experiments conducted at intervals of 20 to 30 minutes to determine the relaxation time T_2_. Currently, magnetic resonance imaging (MRI) is conducted every 30 minutes. The analysis uncovers the existence of three distinct water phases during the soybean infiltration process: bound water denoted as T_21_, sub-bound water represented by T_22_, and free water indicated as T_23_. The evolution of these phases unfolds as follows: bound water T_21_ displays a steady oscillation within the timeframe of 0 to 400 minutes; sub-bound water T_22_ and free water T_23_ exhibit a progressive pattern characterized by a rise-stable-rise trajectory. Upon scrutinizing the magnetic resonance images, it is discerned that the soybean infiltration commences at a gradual pace from the seed umbilicus. The employment of LF-NMR technology contributes significantly by affording an expeditious, non-destructive, and dynamic vantage point to observe the intricate motion of water migration during soybean infiltration. This dynamic insight into the movement of water elucidates the intricate mass transfer pathway within the soybean-water system, thus furnishing a robust scientific foundation for the optimization of processing techniques.

## Introduction

Soybean, a member of the legume family, boasts an abundance of essential nutrients, boasting approximately 40% protein content and 25% fat composition [1]. Within the realms of agriculture and the food industry, soybean assumes a pivotal role as a paramount agricultural commodity and a distinguished wellspring of high-quality plant protein [2]. For example, soybeans are a key ingredient in a myriad of food products, ranging from soymilk and tofu to dried soybeans, tofu skin, and tofu dregs [3–5]. However, it should be noted that elevated levels of moisture content can cause unfavorable consequences, such as mold proliferation, shifts in enzyme activity, and a decrease in the quality integrity of soybeans during their storage and processing phases [6]. Consequently, exploring effective soybean drying techniques and gaining a profound understanding of the dynamic changes in moisture during the soybean infiltration process are crucial for ensuring product quality and sustained supply.

Soybean infiltration and subsequent soybean drying represent sequential stages within the sphere of later-stage soybean storage and processing [7, 8]. Infiltration constitutes a preparatory phase antecedent to the execution of drying, in which soybeans undergo water absorption to facilitate optimal moisture infusion, thus making them suitable for subsequent drying procedures. The state of soybean moistening fundamentally underpins the efficacy of ensuing drying processes [9, 10]. A well-executed infiltration process can result in a more even distribution of moisture within soybeans, thereby preventing excessive gradients between internal and external moisture levels during the drying process, which could otherwise lead to deterioration in quality. This, in turn, mitigates the risk of disproportionate internal and external moisture gradients materializing during the drying trajectory, thus preventing quality deterioration [11]. Consequently, by a deep understanding of the dynamic oscillation in moisture content during the soybean moistening trajectory, informed curation and optimization of drying methodologies can be prudently steered. By comprehensively understanding the dynamic fluctuations in moisture during the soybean infiltration process, a scientific foundation is laid for optimizing the soybean drying process and ensuring the quality and stability of the end product.

Several techniques are available to study the phenomenon of moisture movement, including specific gravity, cross-section, radiofax, X-ray analysis, and Nuclear Magnetic Resonance (NMR) [12–14]. However, specific gravity, cross-section, radiofax, and X-ray analysis techniques are destructive and invasive to varying degrees and do not consistently detect changes in moisture in the sample [15]. The NMR technique, as an emerging analytical test, has achieved great success in the fields of medicine [16], biology [17], and the food industry [18], with advantages such as non-invasiveness, rapidity, and high efficiency. NMR is a physical phenomenon in which nuclear magnetic resonance occurs by applying radio frequency pulses (RF) to a spinning atomic nucleus that is in a static magnetic field B_0_, causing the H protons in it to be excited [19, 20]. Low-field Nuclear Magnetic Resonance (LF-NMR) technique is a new technique for NMR applications, which has the advantages of being rapid, non-destructive, and non-invasive, requiring fewer samples, and acquiring data in real-time [21–23]. Analyzing the LF-NMR signals and observing the NMR images, provides an intuitive reference for the study of the content of water [24–26], oil [27–29], and other components as well as the dynamic change process, and it can be used to determine the content of water and oil at the same time, as well as the content of different parts of water based on the differences in water mobility [30, 31]. On the basis of this premise, the current investigation employed LF-NMR as an innovative detection modality. In this study, both LF-NMR and its associated imaging technology were harnessed to scrutinize the soybean water-mobilization process from an innovative standpoint. Through the analysis of LF-NMR signals, the alterations in water content within soybean seeds after varying soaking durations were investigated. Concurrently, the shifts in water distribution within the soybean seeds were dynamically visualized using magnetic resonance images, thereby effectively showcasing the kinetic progression of soybean wetting. This study continuously monitors the moisture change in the soybean wetting process, which provides an intuitive reference basis for the study of moisture change in the soybean wetting process, provides a scientific tool for the control of the moisture threshold in production practice, and also provides a theoretical basis for determining the target moisture content in the soybean drying process, as well as parameters such as the drying temperature.

The contributions of this work are summarized below.

- The utilization of LF-NMR technology allows for swift, non-destructive, and dynamic visualization of internal water absorption within soybeans, facilitating a comprehensive comprehension of water migration during the soybean infiltration process. This contributes to the understanding of the mass-transfer pathway of water movement in the bean-water system, and thus to the understanding of the complex morphological structure of legumes.
- LF-NMR technology for soybean processing in the infiltration process provides rapid and non-destructive visualization of the technical means.
- Soybean infiltration is one of the most commonly used and important pre-processing procedures. The application of LF-NMR effectively defines complete infiltration and germination according to the water content of different components. This approach provides a scientific foundation for moisture control and optimization of processing techniques.

## Materials and methods

The present study was conducted using a carefully selected array of experimental materials and instruments to ensure rigorous and accurate investigations. The following components were integral to the experimental framework.

### Experimental materials

“Qingtian” brand soybeans were procured from a commercial supermarket, with a production date of June 28, 2023. They originate from Xingtai City, Hebei Province, China. Distilled water was employed for the experiment procedures.

### Experimental instruments

**Fig 1:**
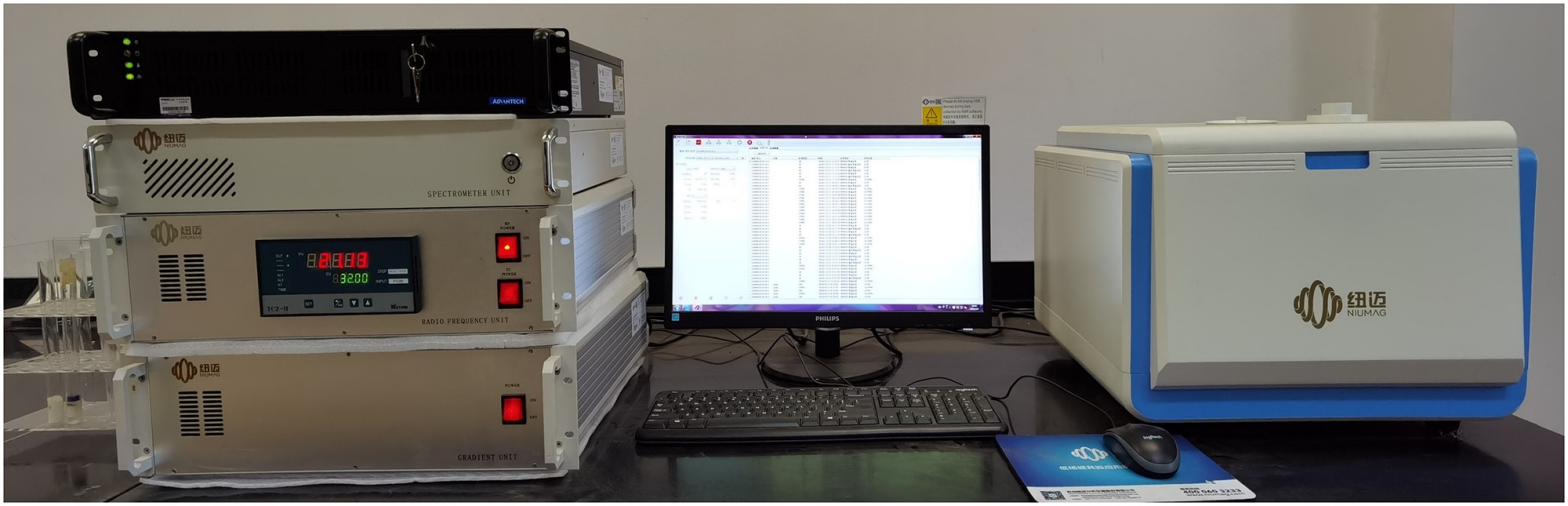
Experimental instruments.

The experimental equipment included an NMI20-015V-I nuclear magnetic resonance analyzer (Fig 1), sterile Petri dishes, special magnetic resonance test tubes, sterile gauze, and sterile forceps. The NMR analyzer’s main function was magnetic resonance imaging and relaxation time analysis of water-containing samples. Its resonance frequency (SFOI) was set to 20.826112 MHz. The magnetic field strength was 0.5T±0.08T and the coil diameter was 15 mm. The equipment was equipped with a thermostat to control the temperature at 32±0.01℃ to ensure the accuracy of experimental results.

### Experimental parameters

#### Parameter setting of CPMG sequence in T_2_ relaxation test

The soybeans were equilibrated to room temperature to measure the relaxation time T_2_ using the LF-NMR analyzer to generate a Carr–Purcell–Meiboom–Gill (CPMG) pulse sequence. The measurement conditions were as follows. The 90-degree RF pulse width (P1) was set to 16 μs, the 180-degree RF pulse width (P2) was set to 33.04 μs, the echo time (TE) was set to 0.200 ms, the echo number (NECH) was set to 18000, and the repeated scanning time (NS) was set to 8. The T_2_-FitFrm software was used to execute the fitting of T_2_ values.

#### T_1_ weighted imaging parameter setting of Low-field MRI

MRI was performed with the same LF-NMR analyzer and the Inversion Recovery (IR) sequence. The soybean was placed in the center of the radio frequency (RF) coil to collect the signal and obtain a T_1_-weighted image. The main parameters were configured as follows. The sampling repetition time (TR) was set to 500 ms, the echo time (TE) was set to 20 ms, the matrix size was set to 192 × 256, and the field of view (Fovx and Fovy) was set to 80 mm.

### Experimental principles and methods

#### Sample preparation

A meticulous sample preparation process was undertaken to establish a solid foundation for the subsequent experimental investigations. The following steps outline the comprehensive sample preparation process conducted for this study:

Experimental Design: The experiment incorporated the establishment of a control group and a experimental group.

Control Group: The control group consists of two Petri dishes, labeled #1 and #2. Each dish contains one soybean. Subsequently, distilled water was introduced to immerse the soybeans thoroughly.

Experimental Group: The experimental group comprises two sets of Petri dishes, labeled #1 and #2. The #1 Petri dishes were designated for the analysis of spin-spin relaxation time (T_2_), while the #2 Petri dishes were allocated for nuclear magnetic resonance T_1_ weighted imaging. In #1, there were 22 Petri dishes, and in #2, there were 13 Petri dishes. Each dish contains one soybean immersed in distilled water, allowing the soybeans to soak thoroughly in water at 25 degrees Celsius. In dishes 1-12 of #1, the soaking time intervals were 20 minutes, while in dishes 13-22 of #1, the soaking time intervals were 30 minutes. The soaking time intervals for soybeans in the dishes of #2 were 0.5 hours.

#### Experimental principles

Soybeans belong to a relatively intricate multi-component system, and within the context of the soybean infiltration process, water manifests itself in at least three distinct forms within the soybeans: bound water, sub-bound water, and free water. NMR methodology quantifies the relaxation time T_2_ associated with these water phases, thereby facilitating the observation of their respective binding states. Variations in relaxation time T_2_ can elucidate the mobility patterns of water molecules, thus providing information on the migratory dynamics of water within soybeans [32].

To accurately ascertain the authentic T_2_ relaxation characteristics of soybean infiltration through magnetic resonance signals, it is essential to endeavor to mitigate the impact of external constant magnetic field inhomogeneity on T_2_ relaxation during measurements. This objective is achieved through the employment of the Carr-Purcell-Meiboom-Gill (CPMG) pulse sequence, which is explicitly designed to counteract this influence.

**Fig 2:**
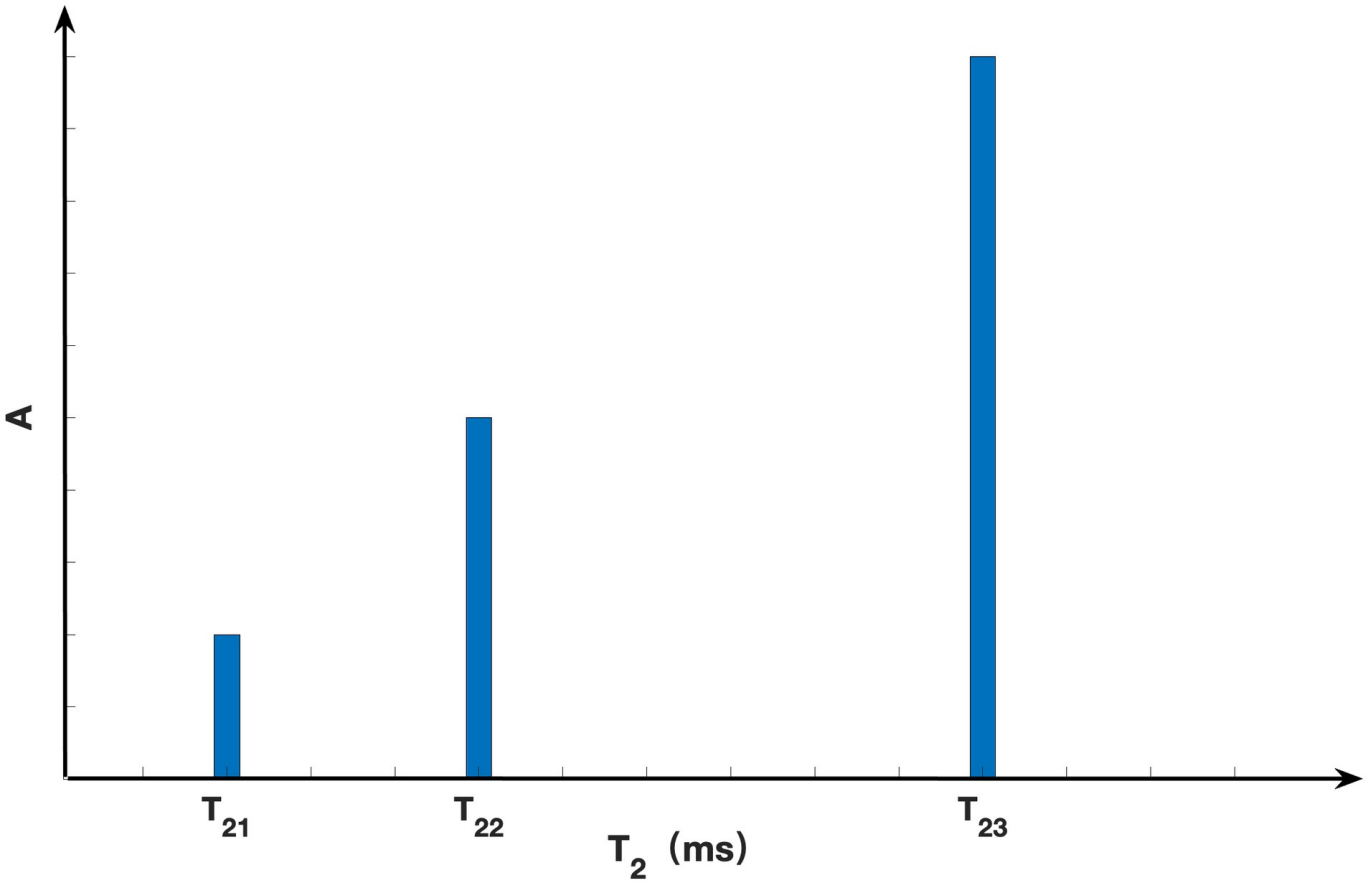
Distribution of spin-spin relaxation time T_2_ of three-component model.

In the context of this experiment, the analysis relies upon the utilization of the three-component model inherent in the multiple exponential decay framework of the CPGM pulse sequence [33].

$$\begin{array}{c} A=A_{1}e^{-\frac{t}{T_{21}}}+A_{2}e^{-\frac{t}{T_{22}}}+A_{3}e^{-\frac{t}{T_{23}}}, \end{array}$$ (1)

where T_21_, T_22_ and T_23_ signify the spin-spin relaxation time of three distinct components, while A_1_, A_2_ and A_3_ denote the signal amplitudes of these respective components at time t. Furthermore, A represents the overall signal amplitude at time t. The magnitude of the relaxation time T_2_ signifies the intensity of water fluidity, as depicted in Fig 2.

Employing the multi-component model for analyzing the relaxation behavior of protons in samples facilitates the segregation of signal amplitudes and relaxation times of protons about distinct components. Increased water fluidity corresponds to greater water molecule mobility, characterized by higher motion frequencies surpassing the resonance frequency of hydrogen protons. Consequently, this leads to the extension of relaxation time.

Conversely, reduced water flowability restrains water molecules due to their close association with hydrophilic macromolecules. As a consequence, these molecules exhibit slower movement rates, that approximate the Larmor frequency, culminating in shortened relaxation times.

### Low-field NMR method

#### Testing the T_2_ relaxation of the soybeans

The measurement of soybean T_2_ relaxation time is a pivotal experiment within the scope of this study. This test aims to delve into the dynamic changes in moisture distribution within soybeans using LF-NMR technology [34]. The experimental procedure is as follows:

Step 1: Using the CPMG sequence [35] within the NMR analysis software, retrieve the T_2_ value of soybeans within the #1 Petri dish of the control group.
Step 2: After varying the duration of soybean infiltration in the #1 Petri dish of the experimental group, measure the T_2_ value, proton density value, and signal intensity value.

For infiltration durations of up to 4 hours, conduct measurements at 20-minute intervals; for infiltration periods ranging from 4 to 9 hours, perform measurements at 30-minute intervals. For soybeans of different Petri dishes in the control group and experimental group, the measurement of T_2_ relaxation time was repeated three times, and the average value was reported.

#### Low-field MRI T_1_ weighted imaging of the soybeans

Utilizing low-field MRI T_1_-weighted imaging to dynamically observe changes in moisture distribution within soybean seeds, thus illustrating the dynamic process of soybean imbibition. An overview of the experimental procedure is provided as follows:

Step 1: Conduct low-field MRI on soybeans within the Petri dish # 2 of the control group to acquire T_1_-weighted images.
Step 2: After varying durations of soybean infiltration in the #2 Petri dish of the experimental group, transfer the soaked soybeans into test tubes and subject them to low-field MRI using the NMR instrument. Capture T_1_-weighted images at intervals of 0.5 hours throughout the soybean infiltration process.

## Results and discussion

Soybean infiltration denotes the hydrating process of soybeans, typically encompassing two distinct stages [36].

The initial stage involves swelling and water absorption, primarily reliant on soybean colloid and unrelated to soybean metabolism. Within this phase, soybean colloid transitions from a gel-like state to a sol state through water absorption and swelling. This transformation facilitates the extension and restoration of compromised organelles and inactivated polymers present in desiccated soybeans.

The subsequent stage entails gradual water absorption. Following the rapid water uptake in the initial stage, soybean hydration reaches a near-saturation point, leading to heightened cellular expansion pressure. This pressure impedes further water absorption by the cells. Consequently, this phase witnesses the principal metabolic activities of soybeans.

According to NMR principles, free water molecules exhibit a considerably extended T_2_ relaxation time, spanning several hundred milliseconds. Their diminutive size allows for swift movement, significantly surpassing the resonant frequency of ^1^H. Conversely, bound water congregates around hydrophilic macromolecules, constrained by these macromolecules and thus exhibiting sluggish motion. Its motion frequency aligns closely with the Larmor frequency, expediting relaxation and resulting in a notably smaller T_2_ value compared to that of free water.

The relationship between the relaxation time T_2_ of various water-binding states can be summarized as follows:

$$\begin{array}{c} Bound\;water\leq Sub-bound\;water\leq Free\;water \end{array}$$ (2)

The relaxation time T_2_, proton density value, and the signal intensity value of different water phases at various time points are determined by observation of the state of the soybean water and changes over time. Subsequently, the ratio of proton density to signal intensity denoted as the A_2_ value, is calculated, and the results are presented in Table 1.

**Table 1:** Changes of relaxation time T_2_ and proportion A_2_ of three-phase water soaked in soybeans for different periods of time.

Time(min)	T_21_(ms)	T_22_(ms)	T_23_(ms)	A_21_(%)	A_22_(%)	A_23_(%)
0	0.301	7.40	98.8	36.9	0.825	62.1
20	0.367	8.50	130	16.4	37.9	45.5
40	0.401	12.0	180	11.7	55.1	33.1
60	0.435	12.0	211	5.10	67.0	27.8
80	0.435	13.0	231	2.48	81.6	15.9
100	0.471	14.1	240	2.88	87.3	9.80
120	0.511	15.3	261	4.32	87.8	7.82
140	0.314	16.6	303	6.19	87.5	6.24
160	0.471	18.0	333	6.27	87.9	5.79
180	0.401	18.0	333	7.28	88.5	4.14
200	0.471	19.5	361	5.84	90.2	3.85
220	0.435	19.5	380	6.28	90.3	3.40
240	0.471	21.2	391	5.59	90.3	4.01
270	0.471	21.2	391	5.76	90.3	3.84
300	0.437	21.2	391	6.51	90.6	2.84
330	0.471	21.2	391	6.79	90.9	2.23
360	0.511	24.9	403	6.49	90.9	2.57
390	0.571	27.0	424	7.69	89.9	2.32
420	0.766	27.0	424	6.93	91.4	1.65
450	0.601	29.3	499	7.00	91.5	1.43
480	0.707	29.3	499	7.83	90.7	1.41
510	0.601	34.4	587	7.82	91.0	1.14
540	1.149	34.4	587	7.16	91.2	1.57

**Fig 3:**
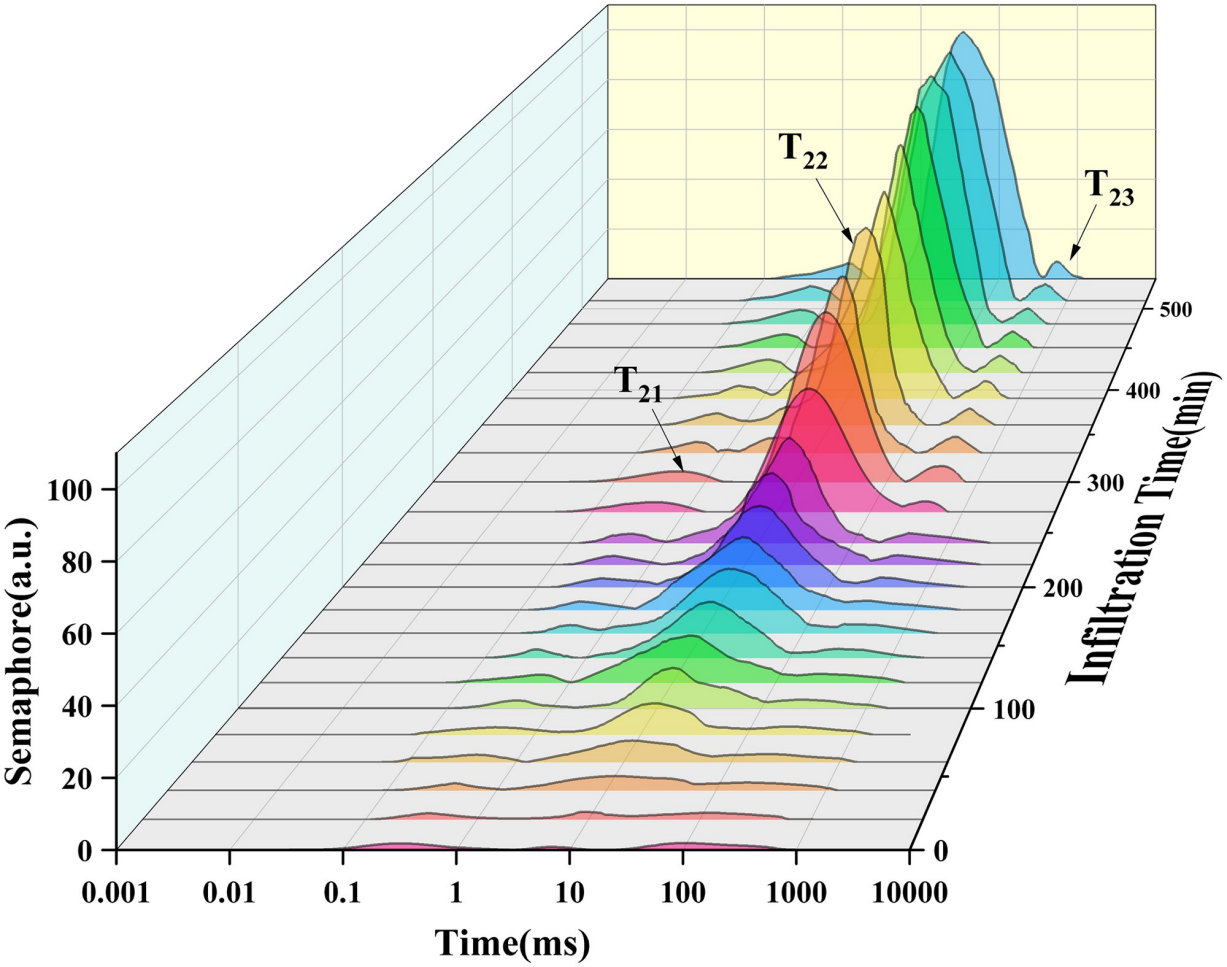
T_2_ relaxation diagram of soybean infiltration process.

Fig 3 illustrates the T_2_ relaxation curve of soybeans. The x-axis represents relaxation time, while the y-axis denotes the relative water content in distinct stages. The highest point on each peak signifies the T_2_ relaxation, and the area beneath the peak signifies the content of the relaxation component. Each peak corresponds to a specific type of water, thereby showcasing the evolving water migration within soybeans during the soaking process at varying immersion durations.

As depicted in Fig 3, it is evident that before infiltration, soybeans predominantly contain bound water, as indicated by the T_21_ absorption peak. Following the infiltration process, the presence of free water rises, signifying the infiltration of water into soybean tissue. With time during infiltration, the volume of flowing water augments, leading to the emergence of two distinct water peaks (T_22_, T_23_) within the soybeans. These peaks correspond to sub-bound water and free water, respectively, and exhibit a shift toward longer relaxation times as the duration of infiltration is extended.

### Analysis of water binding state during soybean infiltration

The data presented in Table 1 reveals that water molecules during soybean infiltration undergo interaction with soybean macromolecules, resulting in the formation of three distinct water phases: bound water, sub-bound water, and free water. These phases exhibit varying relaxation time rates and fluidity characteristics.

The spin-spin relaxation time for bound water, characterized by limited fluidity, is denoted as T_21_ and falls within the 0.3 to 1.2 milliseconds range. Sub-bound water, exhibits higher fluidity than bound water, with a spin-spin relaxation time denoted as T_22_, which ranges between 10ms and 40ms. The most favorable fluidity is observed in free water, with a spin-spin relaxation time represented as T_23_, ranging from 100 ms to 1000 ms.

#### Analysis of T_21_ curve change

**Fig 4:**
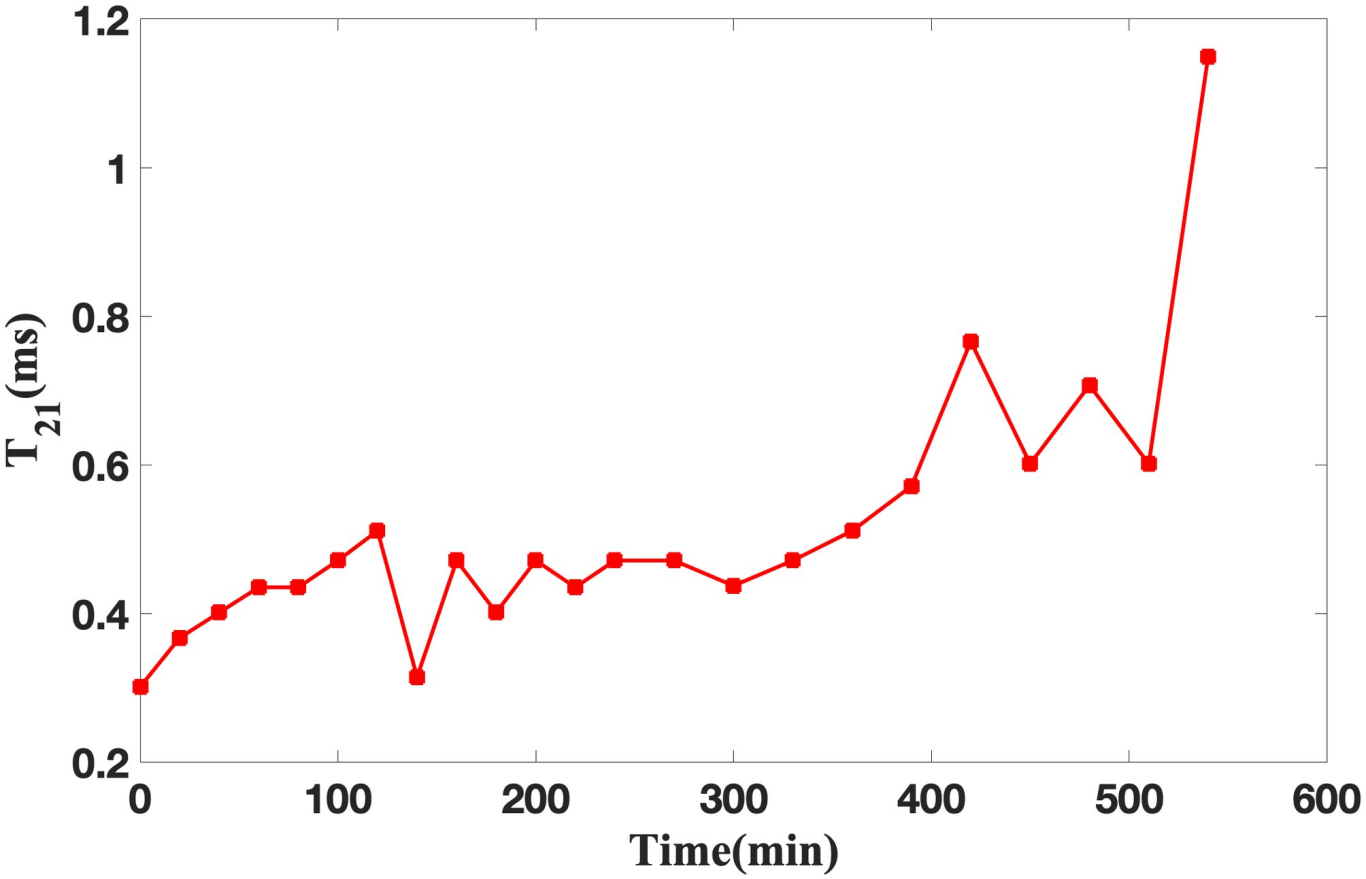
Changes of T_21_ during soybean infiltration.

Fig 4 illustrates the variation in the relaxation time T_2_ associated with bound water throughout the soybean infiltration process. As depicted in Fig 4, T_21_ exhibits minimal discernible alteration. T_21_ represents the bound water that is intimately associated with the macromolecules within soybeans and is the residual water following soybean drying. During the 300-minute soybean soaking period, T_21_ experiences intermittent fluctuations while maintaining stability, exhibiting an overall ascending trajectory, the value of T_21_ is in the range of 0.3 to 0.47 ms. Upon the completion of 400 minutes of soybean soaking, T_21_ experiences a sudden upsurge, increasing from 0.57 ms to 1.15 ms. This phenomenon signifies the initiation of macromolecule synthesis and transformation within soybeans, accompanied by a reduction in the firmness of the binding between bound water and macromolecules.

#### Analysis of T_22_ and T_23_ curve change

**Fig 5:**
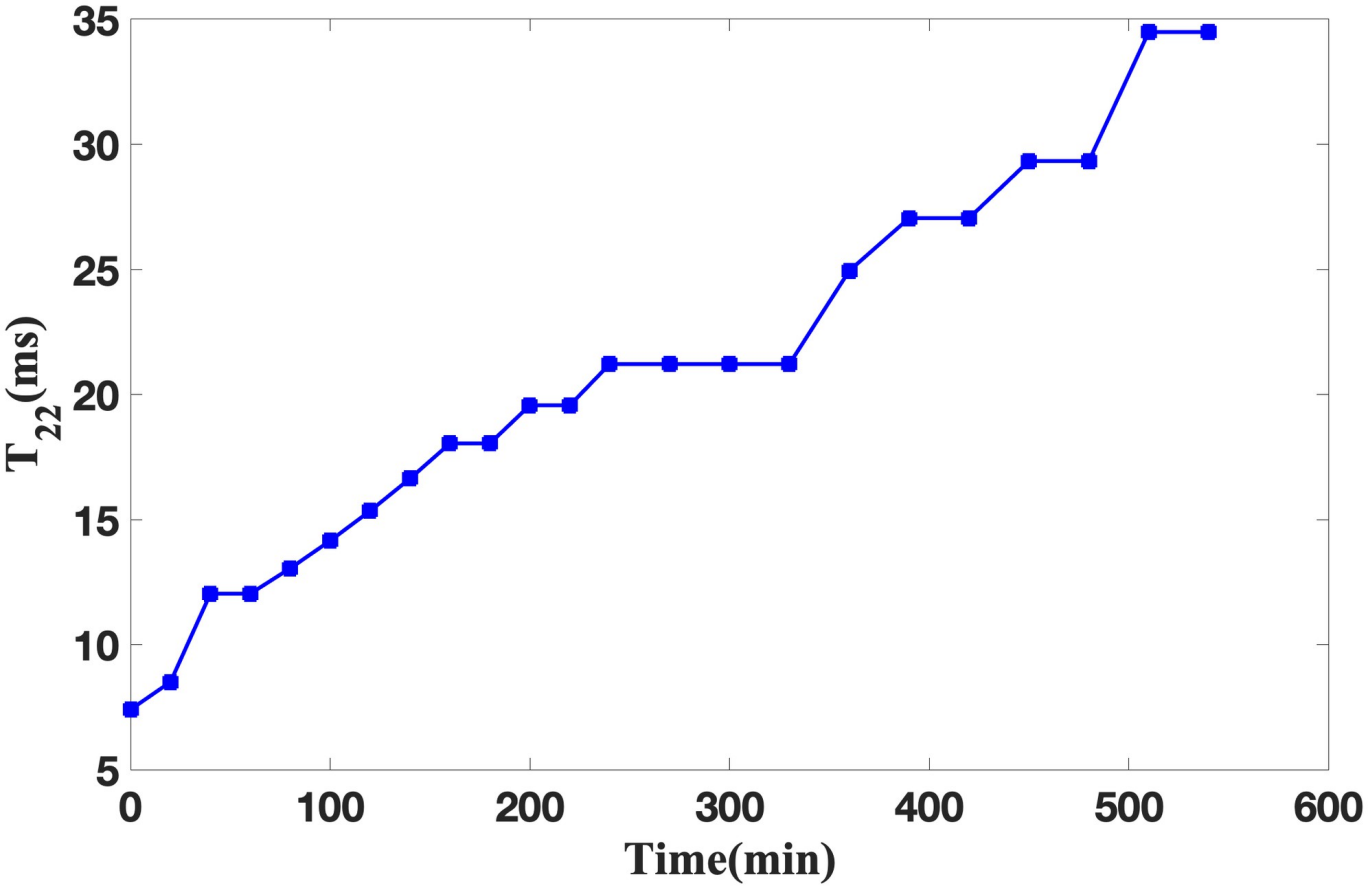
Changes of T_22_ during soybean infiltration.

**Fig 6:**
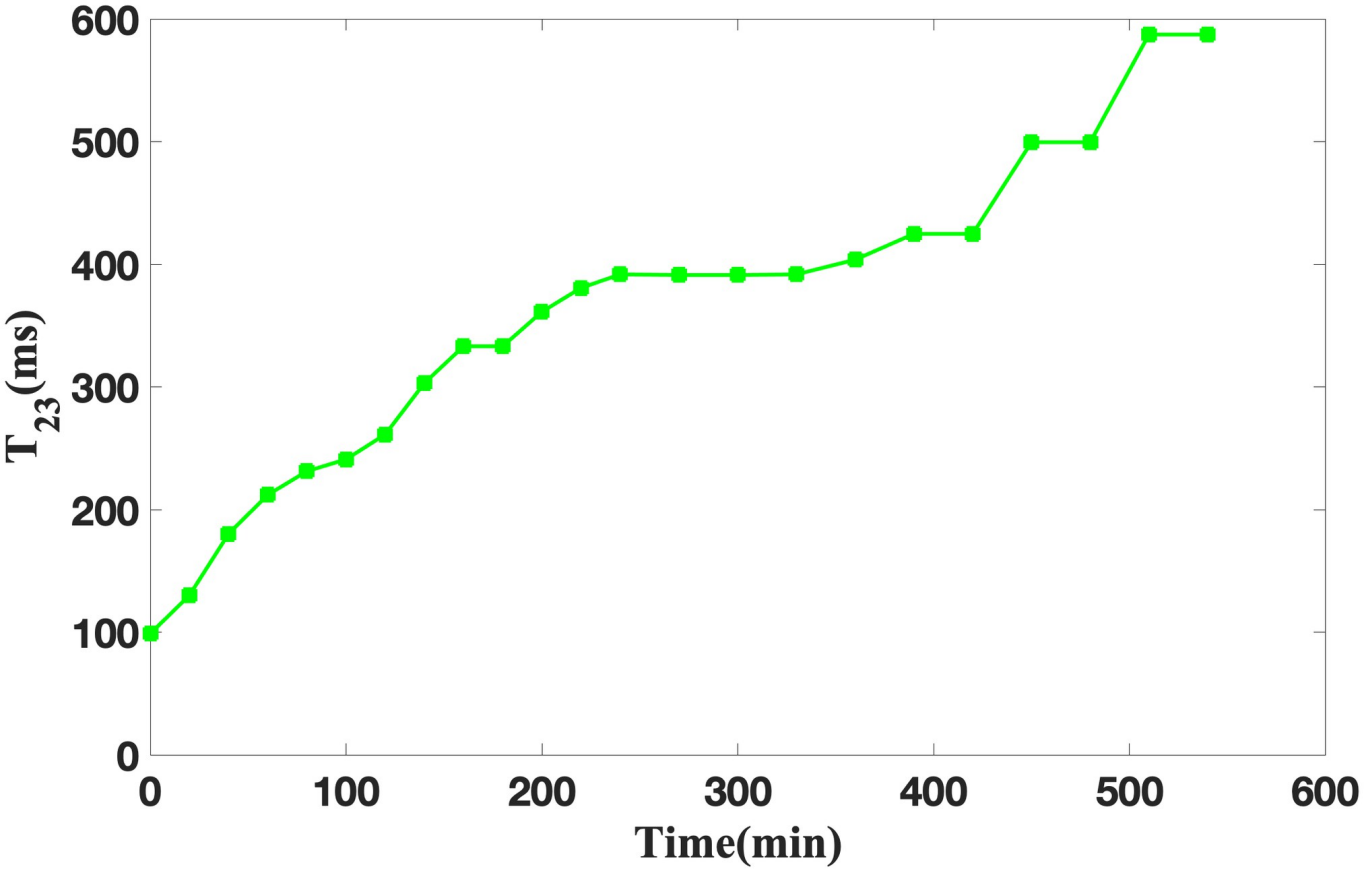
Changes of T_23_ during soybean infiltration.

The observations from Figs 5 and 6 reveal that during the soybean infiltration process, the ingress of water molecules into the soybeans manifests as free water, whereas water molecules binding with hydrophilic macromolecules within soybeans manifest as sub-bound water. When the soaking duration is below 200 minutes, both the relaxation time T_22_ of sub-bound water and T_23_ of free water exhibit substantial increments, T_22_ increased from 7.4 ms to 19.56 ms, while T_23_ increased from 98.85 ms to 361.23 ms. This phenomenon signifies the commencement of soybean hull softening and water absorption, inducing soybean activation and breaking the dormancy phase. Subsequently, approximately after 400 minutes of soaking, concurrent with soybean activation, water begins to participate in the activation of existing enzymes or the synthesis of novel enzymes. The fluidity of water in soybean is enhanced, which is reflected by the evident elevation in T_22_ and T_23_, T_22_ increased from 27.05 ms to 29.33 ms, while T_23_ increased from 424.76 ms to 499.45 ms. Beyond 500 minutes of soaking, T_22_ and T_23_ stabilize, maintaining at 34.49 ms and 587.28 ms, respectively, indicating the cessation of soybean activation. This milestone suggests that the soybeans have undergone thorough infiltration.

Table 2 illustrates the application of linear regression analysis, utilizing T_22_ (ms) as the independent variable and A_22_ (%) as the dependent variable. As depicted in Table 2, the resultant model equation is: A_22_ (%) = 42.364 + 1.906 × T_22_ (ms), and the corresponding model R-square value stands at 0.431. This signifies that T_22_ (ms) is capable of elucidating approximately 43.1(%) of the variation in A_22_ (%). Subsequently, subjecting the model to an F-test reveals its successful compliance with the test (F = 15.908, p = 0.001 < 0.05), indicative of the influential role of T_22_(ms) on A_22_ (%). Further detailed analysis corroborates this finding: the regression coefficient of T_22_ (ms) stands at 1.906 (t = 3.989, p = 0.001 < 0.01), signifying a statistically significant positive impact of T_22_ (ms) on A_22_ (%). These results collectively assert that all instances of T_22_ (ms) exert a notable and positive influence on A_22_ (%).

**Table 2:** T_22_ liner regression analysis results.

Linear regression analysis results (n = 23)									
	Non-standardized coefficient		Standardization factor	t	p	VIF	R^2^	Adjustment R^2^	F
	B	Standard error	Beta						
Constant	42.364	10.288	-	4.118	0.000**	-	0.431	0.404	F (1,21) = 15.908 p = 0.001
T_22_ (ms)	1.906	0.478	0.657	3.989	0.001**	1.000			
Dependent variable: A_22_ (%)									
D-W value: 0.309									
* p < 0.05 ** p < 0.01									

Table 3 presents the result of a linear regression analysis employing T_23_ (ms) as the independent variable and A_23_ (%) as the dependent variable. As indicated in Table 3, the resultant model equation takes the form: A_23_ (%) = 45.472 − 0.099 × T_23_ (ms), accompanied by a model R-square value of 0.647. This signifies that T_23_ (ms) possesses the capacity to expound upon approximately 64.7(%) of the variability observed in A_23_ (%). Subsequently, subjecting the model to an F-test corroborates its successful passage (F = 38.463, p = 0.000 < 0.05), affirming the impact of T_23_ (ms) on A_23_ (%). Detail analysis further confirms this result: The regression coefficient of T_23_ (ms) stands at -0.099 (t = -6.202, p = 0.000 < 0.01), signifying a statistically significant positive influence of T_23_ (ms) on A_23_ (%). These findings collectively establish that all instances of T_23_ (ms) wield a significant and positive influence on A_23_ (%).

**Table 3:** T_23_ liner regression analysis results.

Linear regression analysis results (n = 23)									
	Non-standardized coefficient		Standardization factor	t	p	VIF	R^2^	Adjustment R^2^	F
	B	Standard error	Beta						
Constant	45.472	5.936	-	7.661	0.000**	-	0.647	0.630	F (1,21) = 38.463 p = 0.001
T_23_ (ms)	-0.099	0.016	-0.804	-6.202	0.000**	1.000			
Dependent variable: A_23_ (%)									
D-W value: 0.263									
* p < 0.05 ** p < 0.01									

Observations and statistical analyzes derived from alterations in the T_22_ and T_23_ curves reveal a significant correlation between T_2_ variations and the fluidity of water molecules. This correlation aids in comprehending the intricate migration dynamics of water molecules within soybeans. The utilization of T_2_ measurement through low-field NMR constitutes a direct and effective approach to gauging the fluidity of water molecules.

### A_2_ and the variation of water ratios in three phases

Throughout the process of soybean infiltration, water migration unfolds as a complex phenomenon. Water molecules ingress the soybeans and initiate interactions with macromolecules, notably proteins. This interaction gives rise to three distinct water phases: bound water, sub-bound water, and free water. Through the analysis of the A_2_ parameter, insights into the temporal evolution of water ratios across these phases can be garnered. This analytical approach facilitates the determination of the optimal soaking duration required for soybeans to achieve the highest water absorption efficiency.

**Fig 7:**
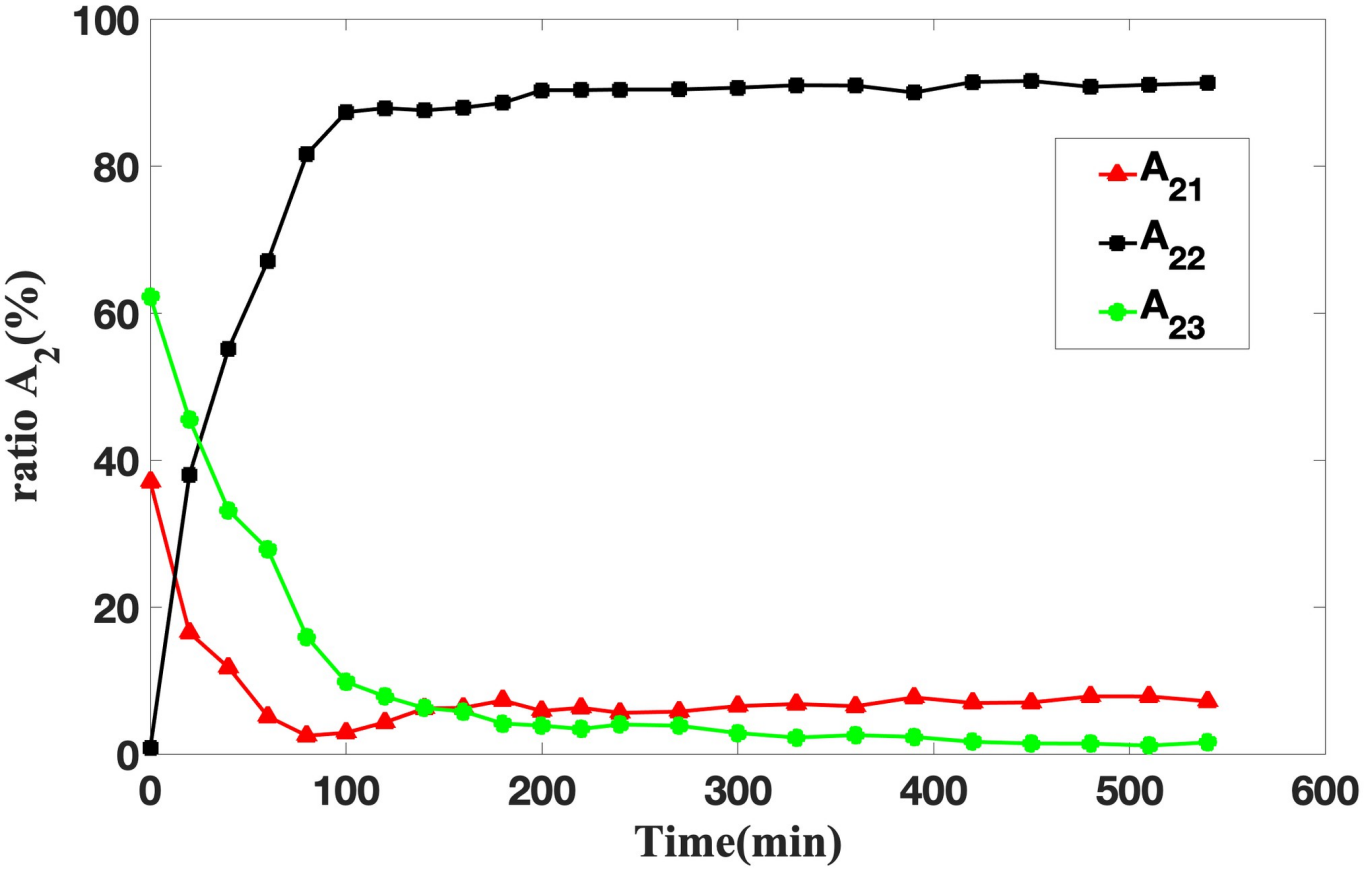
The curve of the ratio A_2_ changing with time.

Fig 7 illustrates the temporal variations in the A_2_ water ratio for the three distinct phases. The following observations can be made.

(1) Initial Dry State: Initially, when soybeans are in a dry state, the proportion of bound water (A_21_) is approximately 36%, sub-bound water (A_22_) is around 0.8%, and free water (A_23_) constitutes approximately 62% of the total water content. This composition suggests that water in soybeans predominantly consists of bound and free water in this dry state.
(2) Early Infiltration Phase: In the early stages of soybean infiltration, both A_21_ and A_23_ experience rapid decreases within the first 180 minutes. Specifically, A_21_ decreases to 2.48% after 80 minutes, while A_23_ drops to 9% at the 100 minute mark. Simultaneously, A_22_ exhibits a swift increase, reaching 87% after 100 minutes of soaking. This shift in water distribution underscores the cessation of dormancy in soybeans during this time.
(3) Continued Infiltration Phase: During the continuous soak of soybeans for 180 to 420 minutes, A_22_ remains stable, maintaining at 90.3% to 91.42%, A_21_ shows a gradual upward trajectory, increasing from 5.85% to 6.93%, and A_23_ experiences a slow decline, decreasing from 3.85% to 1.65%. This stage coincides with the soybeans actively absorbing water and initiating metabolic activities, such as activation.
(4) Germination Phase: Beyond 420 minutes of soaking, the proportions of water in the three phases stabilize. At this juncture, the preparatory activities prior to soybean germination have concluded, manifesting the onset of the germination process.

### T_1_ weighted image analysis of Low-field MRI

**Fig 8:**
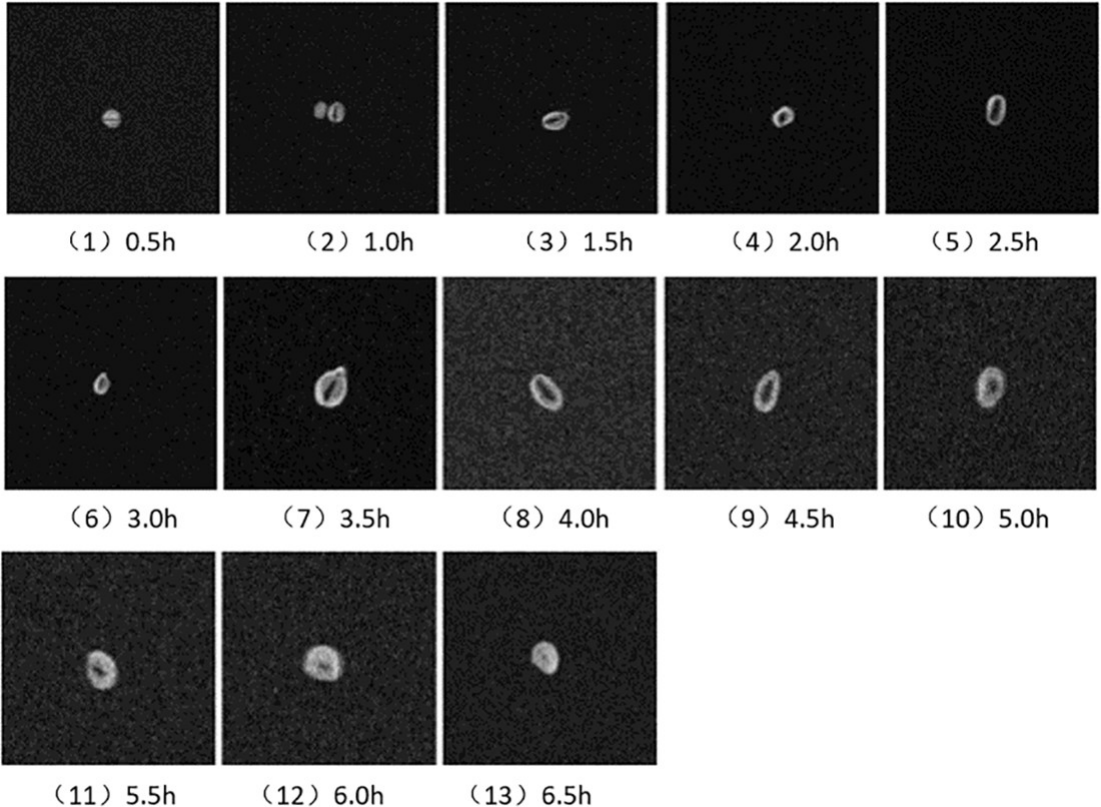
Soybean infiltration imaging.

Low-field MRI scans are conducted on the soybeans immersed in the #2 Petri dish within the experimental group for varying time intervals. T_1_-weighted images are acquired at 30-minute intervals, as depicted in Fig 8.

Free water consists of smaller water molecules that exhibit a heightened thermal motion frequency, leading to diminished energy exchange efficiency between protons and their surrounding environment. The proton motion frequency in free water is lower than the Larmor precession frequency, thereby resulting in an extended spin-lattice relaxation time (T_1_). Biological macromolecules, such as proteins, undergo gradual movement, and their proton motion frequency is significantly removed from the system’s resonance frequency, consequently exhibiting a lengthier T_1_. Nonetheless, the differential between proton motion frequency and the Larmor precession frequency in free water is more substantial than that of biological macromolecules, culminating in an extended T_1_ for free water in contrast to larger molecules.

In the context of cholesterol and adipose tissue, their vibration frequency aligns with the typical MRI field strength’s Larmor precession frequency, resulting in markedly abbreviated T_1_ times. Bound water’s motion frequency closely corresponds to the Larmor precession frequency, thereby conferring a notably truncated T_1_ for bound water as well. Employing low-field MRI to analyze different T_1_ tissues yields Free Induction Decay (FID) signals of varying intensities, which manifest distinct grayscale distinctions in T_1_-weighted images. Tissues characterized by prolonged T_1_ engage in a sluggish longitudinal magnetization recovery, culminating in diminished signal intensity and generating darker images.

In the experiment, the soybean samples subjected to nuclear magnetic resonance imaging comprise three distinct constituents: soaked free water, soybean macromolecular components (like proteins, starch, etc.), and bound water absorbed by the soybeans. The T_1_ of bound water is exceptionally brief, causing the magnetization vector to pivot toward the xOy plane post-90-pulse excitation, resulting in the most substantial amplitude and generating the strongest FID signal. Consequently, this segment appears the brightest within MRI images. Conversely, free water exhibits the longest T_1_, leading the magnetization vector after a 90-pulse excitation to align minimally with the xOy plane, subsequently yielding the feeblest FID signal and presenting as a darker portion within the MRI image. The T_1_ of macromolecular substances in soybeans is proximate to the pulse repetition time (TR), resulting in an FID signal intensity lower than that of free water. This material manifests as a blackened region in the MRI image.

In Fig 8, the region of greatest brightness corresponds to bound water absorbed by the soybeans, the relatively brighter area corresponds to free water, and the darkest segment represents the presence of macromolecular substances. Observing Fig 8(1) and 8(2), it becomes evident that free water enters the soybeans via the navel of the soybean hull. Fig 8(3)–8(9) depict a discernible increase in both the total water content and the proportion of bound water during the soybean infiltration process. As the infiltration time progresses, water permeates deeper into the inner layers, and the T_1_-weighted image vividly displays a state of wetness. Notably, Fig 8(10) portrays that at the 5.0-hour mark of wetting, moisture permeates and accumulates within the interior, indicative of a diffusion trend. Furthermore, Fig 8(13) illustrates that after soaking for 6.5 hours, the soybeans commence a gradual saturation process, moving towards germination.

## Conclusion

By analyzing the LF-NMR signals, this study approached the soybean infiltration process from a novel perspective, unraveling the intricate binding dynamics of water within soybeans. The T_2_ relaxation spectrum unveiled three distinct peaks, signifying the segmentation of soybean internal water into three categories: bound water, sub-bound water, and free water. During the initial 0-180 minutes of infiltration, both the proportion of bound water and sub-bound water exhibited a rapid decline, indicative of external water infiltration into the soybean. As the infiltration time progressed to 180-420 minutes, the proportion of sub-bound water stabilized, while the proportion of bound water underwent a gradual increase, and the proportion of free water showed a gradual decrease. This phase marked the commencement of soybean water absorption, accompanied by activation and metabolic activities. With infiltration time surpassing 420 minutes, the proportions of the three water phases remained stable, denoting the completion of preparatory activities before germination for the soybeans.

The utilization of low-field MRI offers a rapid, non-invasive, and dynamic approach to observing the internal water uptake of soybeans. This technique proves valuable in comprehensively capturing water migration dynamics during the infiltration process and dying process, elucidating the routes of mass transfer within the bean-water system, and discerning the intricate morphological configuration of soybeans. LF-NMR serves as an expeditious and non-destructive visual tool, furnishing insights into the soybean infiltration process. Moreover, it enables the determination of distinct components’ water content based on differing fluidity, thereby furnishing a scientific foundation for controlling the critical point of moisture in production practice and establishing a theoretical basis for determining the target moisture content during the soybean drying process.

This study has achieved success by sufficiently exploring the moisture distribution during the dried soybean infiltration processes, but there is still room for improvement. First, there are differences in moisture content and distribution of soybeans at different ages. Second, soybeans’ moisture content and distribution at different temperatures are also different. To this end, we will carry out the future works on studying the soybean infiltration process from two aspects. First, we will collect diverse soybean samples from different ages to explore the potential relationship between the moisture content and distribution of soybeans and soybean age. Second, we will immerse soybean samples of the same age in distilled water at different temperatures to explore the effect of temperature on the moisture content and distribution of soybeans.
